# Criteria for Advanced Prosthetic Foot Prescription: Rationale, Design, and Protocol for a Multisite, Randomized Controlled Trial

**DOI:** 10.2196/45612

**Published:** 2023-04-04

**Authors:** Jason T Maikos, Brad D Hendershot, Alison L Pruziner, Michael J Hyre, John M Chomack, Samuel L Phillips, Jeffrey T Heckman, Alexis N Sidiropoulos, Christopher L Dearth, Leif M Nelson

**Affiliations:** 1 Veterans Affairs New York Harbor Healthcare System New York, NY United States; 2 Walter Reed National Military Medical Center Bethesda, MD United States; 3 Extremity Trauma and Amputation Center of Excellence Falls Church, VA United States; 4 Uniformed Services University of the Health Sciences Bethesda, MD United States; 5 National Veterans Sports Programs and Special Events Department of Veterans Affairs Washington, DC United States; 6 The Narrows Institute for Biomedical Research and Education, Inc Brooklyn, NY United States; 7 James A Haley Veterans’ Hospital Tampa, FL United States; 8 Veterans Affairs Puget Sound Healthcare System Seattle, WA United States; 9 Regional Amputation Center Veterans Affairs Puget Sound Healthcare System Seattle, WA United States; 10 Department of Rehabilitation Medicine University of Washington Seattle, WA United States

**Keywords:** limb loss, amputation, prosthetic prescription, prosthetic ankle-foot devices, lower extremity

## Abstract

**Background:**

The prescription of prosthetic ankle-foot devices is often based on the professional judgment of the limb loss care team or limited evidentiary research. Current prosthetic research efforts have focused on the design and development of prosthetic devices rather than on understanding which devices are the most appropriate to prescribe. This investigation will evaluate biomechanical, functional, and subjective outcome measures to help determine the optimal prescription parameters of prosthetic ankle-foot devices.

**Objective:**

This study aims to develop evidence-based guidelines for limb loss care teams for the appropriate prescription of commercially available prosthetic ankle-foot devices to improve function and satisfaction.

**Methods:**

This investigation will be a multisite, randomized, crossover clinical trial targeting the enrollment of 100 participants. Participants will use 3 different types of prosthetic devices (energy storing and returning, articulating, and powered) in random order. Participants will be fitted and trained with each device and then separately use each device for a 1-week acclimation period. Following each 1-week acclimation period, participants will be evaluated using several functional measures and subjective surveys. A random subset of participants (30/100, 30%) will also undergo full-body gait analysis, following each 1-week acclimation period, to collect biomechanical data during level ground and incline and decline walking. After all individual device evaluations, participants will be given all 3 prostheses concurrently for 4 weeks of home and community use to capture user preference. Activity monitoring and a guided interview will be used to determine overall user preference.

**Results:**

The study was funded in August 2017, and data collection began in 2018. Data collection is expected to be completed before July 2023. Initial dissemination of results is expected to occur in the winter of 2023.

**Conclusions:**

By identifying biomechanical, functional, and subjective outcomes that are sensitive to differences in prosthetic ankle-foot devices, a benchmark of evidence can be developed to guide effective prosthetic prescription.

**Trial Registration:**

ClinicalTrials.gov NCT03505983; https://clinicaltrials.gov/ct2/show/NCT03505983

**International Registered Report Identifier (IRRID):**

DERR1-10.2196/45612

## Introduction

### Background

More than 1700 service members have experienced combat-related limb loss since the beginning of Operation Iraqi Freedom, Operation Enduring Freedom, and Operation New Dawn [1,2]. Most of these service members are now veterans, and 95% of veterans with limb loss from these conflicts use the Veterans Affairs (VA) Healthcare System within 5 years of separation from active duty [3]. VA has a responsibility to serve this unique population for their lifetime and provide care to an additional 41,000 veterans who have lower limb loss at levels proximal to the ankle [3]. At VA, most major limb amputations have resulted from the increase in aging veterans with dysvascular disease and diabetes mellitus [4]. For those receiving prosthetic ankle-foot devices, providers within the limb loss care teams have >100 commercial devices available for prescription [5]. Unfortunately, there are limited evidence-based guidelines available to aid clinicians in prescribing appropriate componentry. As such, prescription tends to be governed largely by professional judgment of the limb loss care team or available research, which tends to be noncommittal or lacks the guidance required for clinical practice [6].

### Clinical Consensus for Prosthetic Prescription

As prosthetic ankle-foot devices have evolved over the last 2 decades, clinical prescription has remained a complex and challenging process. Comparative studies have been contradictory or noncommittal, which makes evidence-based prescription difficult for clinicians. In 2004, a Cochrane review was conducted to determine whether a clear clinical consensus on prescription criteria could be determined for various prosthetic ankle-foot devices for individuals with lower limb loss [7]. Review of 23 trials concluded that there was insufficient evidence from high-quality comparative studies to develop or establish criteria for the prescription of prosthetic ankle-foot devices. In 2005, Hafner [8] evaluated clinical prescription and the use of prosthetic ankle-foot mechanisms in the literature, again concluding that the available studies had limited applicability for clinical decision-making. The author suggested that future studies that aim to develop guidelines to prescribe prosthetic devices should include a large sample size and standardize the devices based on shared characteristics or mechanical behaviors. In 2012, Schaffalitzky et al [9] gathered expert opinions on the most important outcomes, predictors, and facilitators of lower limb prosthetic prescription and use. The investigators and an expert panel of 21 service providers and users identified 13 outcomes, 19 predictors, and 34 facilitator factors that can contribute to an evidence base for optimal prosthetic prescription. While highlighting the importance of a comprehensive evaluation, including physical, psychosocial, and environmental outcomes, a universal solution was not identified for optimal prosthetic prescription; however, it is unclear whether all users would benefit from a universal prescriptive solution [9]. Finally, a Cochrane review in 2018, which aimed to investigate the evidence from randomized controlled trials of individuals with lower limb loss, found that the scientific literature did not provide sufficient high-quality evidence to allow strong conclusions about the effectiveness of prosthetic intervention [10]; however, many nonrandomized controlled studies were excluded from the analysis.

### Prosthetic Ankle-Foot Device Categories

Despite the lack of scientific guidelines for appropriate prosthetic prescription, energy storing and returning (ESR) ankle-foot devices are the current gold standard for prescription within VA and the Department of Defense (DoD). Individuals with transtibial limb loss using ESR devices have demonstrated faster walking speeds and reduced cost of energy during ambulation compared with those using traditional prosthetic device options, such as the solid ankle cushion heel and stationary attachment flexible endoskeletal feet [11]. Similarly, the use of ESR devices has been shown to improve elastic response [12], reduce the requisite activation for propulsory muscles [13], and improve stair ascent initiation by reducing the time of initial double-limb support [14]. Although there are various types of ESR devices with distinct mechanical features, there remains insufficient evidence to support the prescription of specific prosthetic ankle-foot devices within this overarching classification [15-17]. Moreover, discrete parameters derived from common kinetic and kinematic biomechanical analyses often lack statistically significant differences between these ESR devices [8].

More advanced ESR devices are also commercially available, including passive hydraulic or microprocessor-controlled articulating ESR (ART) ankle-foot devices. Although ART ankle-foot devices have been typically reported to decrease energy return in individuals with transtibial limb loss, there is recent evidence suggesting that current devices can improve ambulation on level ground and on inclines and stairs [15]. For example, De Asha et al [18,19] described improved progression of the center of pressure in a prosthetic ankle-foot device with a hydraulic ankle compared with devices with a fixed attachment in a population of individuals with lower limb loss. Portnoy et al [20] found decreased load at heel strike when using an ESR hydraulic ankle-foot device compared with a conventional ESR device. Fradet et al [21] reported that a microprocessor-controlled ankle-foot device set to adaptive ankle mode improved kinematics during ramp ascent compared with the same device when set in neutral angle mode. This evidence suggests that individuals with lower limb loss who negotiate specific environments may benefit from an ART device.

Commercially available ESR ankle-foot devices with active (ie, powered) plantarflexion have potential biomechanical benefits for the user beyond that of the ESR or ART ankle-foot options. Empower (Ottobock Inc), the only commercially available powered ESR ankle-foot device, functions to replicate the dynamic contractile tissues of the gastrocnemius-soleus complex in individuals with lower limb loss [22,23]. This powered prosthetic ankle-foot device has the ability to normalize ankle power, which can potentially reduce kinetic asymmetries that lead to musculoskeletal imbalances, thus improving physical function [22,24]. Grabowski and D’Andrea [25] noted that individuals with transtibial limb loss had a reduction in peak resultant forces and knee adduction moments in the unaffected limb during level ground walking when using a powered ESR device (iWalk BiOM) versus a passive ESR device. These reductions could potentially limit the risk of secondary musculoskeletal comorbidities, which are common in this population [26].

Despite the noted advances in prosthetic technology, research efforts so far have largely focused on the design and development of prosthetic technology, rather than on understanding which devices are most optimal to be prescribed for individuals with lower limb loss. Moreover, many studies are limited by small sample sizes, the nonstandardization of device characteristics, and the use of outdated components, which collectively reduce the direct applicability of scientific evidence to clinical decision-making [8]. Through a multicentered clinical trial with a large sample size (N=100), standardized categories of prosthetic ankle-foot characteristics, and the evaluation of outcomes both within laboratory and community-based settings, this research investigation will comprehensively evaluate biomechanical, functional, and subjective outcome measures to help aid in determining optimal prescription parameters of prosthetic ankle-foot devices. Health care, according to comparative effectiveness research, is under constant pressure to become more efficient and effective, while reducing overall cost. The lack of evidence-based scientific guidelines to support lower extremity prosthetic prescription is a critical inefficiency. By identifying biomechanical, functional, and subjective outcomes that are sensitive to differences in prosthetic ankle-foot devices, a benchmark of evidence can be developed to guide effective prosthetic prescription.

### Study Objectives

The overarching goal of this investigation is to generate data that offer new knowledge that can be used to improve current clinical practice guidelines and develop new prescription algorithms for prosthetic ankle-foot devices. This information will lead to clinical prescriptions that facilitate the achievement of maximal functional ability and user satisfaction. Criteria will be developed to assist the limb loss care teams in prescribing appropriate, advanced prosthetic ankle-foot devices based on patient-specific needs and goals, in addition to the individual’s current physical abilities. Therefore, the purpose of this investigation is to develop the criteria for prosthetic ankle-foot prescription for veterans and service members with transtibial limb loss. The objectives of this investigation are as follows:

1. To determine the appropriate functional and biomechanical outcome measures to support the prescription of a type or category of prosthetic ankle-foot device for veterans or service members with transtibial limb loss
2. To correlate patient goals and subjective measures with objective data to determine the appropriate prosthetic ankle-foot category that will facilitate the greatest overall function for the user
3. To develop criteria for the appropriate prescription of nonarticulating ESR, ART, and powered plantarflexion ESR ankle-foot devices

## Methods

### Study Overview

This investigation will be a prospective, multicenter study**,** including 4 sites: Veterans Affairs New York Harbor Healthcare System (VANYHHS), Veterans Affairs Puget Sound Healthcare System, James A Haley Veterans’ Hospital (JAHVH), and Walter Reed National Military Medical Center (WRNMMC). Enrollment began in 2018, and data collection is expected to be completed in 2023. A total of 100 participants will be enrolled from the 4 sites. Briefly, participants will use 3 different types of prosthetic ankle-foot devices (refer to device categorization in the following sections) with duplicate sockets. Participants will be fitted and trained with each device and then separately use each ankle-foot device for a 1-week acclimation period. Participants will only use the assigned study device for each acclimation period. Although previously reported acclimation periods for lower extremity prosthetic device studies have ranged from minutes to months depending on componentry [27-30], this study will use a 1-week acclimation period to balance the time that participants need to adjust to the features of each ankle-foot device with the overall duration of their participation. To augment the acclimation period, device-specific training will be provided to ensure that participants meet a minimum standard of performance. Following each 1-week acclimation period, participants will be evaluated in the laboratory using several functional and subjective measures. Furthermore, a subset of participants (30/100, 30%) will be randomly chosen to undergo a full gait analysis to collect biomechanical data during level ground and incline and decline walking using each ankle-foot device. Following the 3-week data collection period, participants will be given all 3 prostheses at the same time for 30 days to use at home and in their community. Step activity monitoring during home-based and community-based use and a final guided interview will be used to determine overall user preference.

### Participants

In total, 100 individuals with unilateral transtibial limb loss will be recruited for this investigation. Inclusion and exclusion criteria are presented in Textbox 1. All participants will consent to participate before any study activity.

**Inclusion and exclusion criteria.**

**Inclusion criteria**

- Veteran, service member, or civilian with unilateral transtibial limb loss
- Uses a prosthesis with a well-fitting socket for a minimum of 1 month
- Must achieve at least modified independence score on the Functional Independence Measure for the 2 mobility items (locomotion and stairs)
- Aged >18 years
- Current use of an energy storing and returning device
- Has a minimum clearance to accommodate all devices

**Exclusion criteria**

- Active wounds, ulcers, or substantial musculoskeletal comorbidities on the intact limb that would impair the ability to participate in all functional outcome measures
- Any comorbidity that results in rapid limb volume changes (eg, end-stage renal disease with dialysis)
- A poorly fitting socket
- Cognitive deficits or mental health pathology limiting an individual’s ability to fully participate in the investigation
- Unable or unwilling to comply with all study visits
- Women who are pregnant or who plan to become pregnant during study activities, determined via self-report
- Body mass greater than product capacity

### Ethics Approval and Informed Consent

This study was approved by the following institutional review boards (IRBs): Veterans Affairs New York Harbor Healthcare System IRB (protocol ID 1603); University of South Florida IRB (IRB study Pro00030555), which is the IRB of record for James A Haley Veterans’ Hospital; Walter Reed National Military Medical Center IRB (protocol ID WRNMMC-2017-0103); and Veterans Affairs Puget Sound Healthcare System IRB (protocol ID 01587). The oversight and protection of human participants was also approved by the US Army Medical Research and Development Command Office of Human Research Oversight (E04076). All participants will provide informed consent before participating in any study activity.

### Ankle-Foot Categorization

In accordance with recommendations from previous literature to standardize the categorization of devices according to specific features [8], the prosthetic ankle-foot devices included in this investigation were systematically selected and categorized at the time of study initiation by a diverse panel of experts in the limb loss field, including prosthetists, physiatrists, biomechanists, physical therapists, and limb loss researchers. The panel considered device structure, componentry, function, and biomechanical properties to create ankle-foot device categories. On the basis of the criteria developed by the expert panel for each group, all ankle-foot devices included in this investigation were placed into one of the following three categories:

1. ESR (nonarticulating)—This group includes any qualifying nonarticulating ESR ankle-foot device that is commercially available. As per the inclusion criteria, participants will use their current device for this category. There are >100 commercially available devices that fit the criteria for this category [5].
2. Active plantarflexion ESR (PWR)—This group will include all commercially available ESR ankle-foot devices with active plantarflexion. Currently, Empower (Ottobock Inc) is the only device classified into the PWR group. No new devices that meet the criteria for the PWR group will be included in this investigation after the commencement of active enrollment. However, it is not anticipated that any future powered device will come to the market during active enrollment.
3. ART—This group includes all current, commercially available options in the K3 Medicare Functional Classification System category that have an articulating ankle and ESR properties. Overall, 14 devices that met the criteria were identified at the time of study initiation (Table 1). No new devices that meet the criteria for the ART group will be included in this investigation after the commencement of active enrollment. If any ART device is replaced by the manufacturer with a new generation during active enrollment, the study expert panel will determine whether the new generation can be considered as a replacement device (if not fundamentally distinct). If the new generation of ART device is considered to be fundamentally different from the study ART device by the expert panel, it will not be included as a study device, to reduce confounding variables during analysis. However, the current generation of ART device used in the investigation will be configured to match patient specifications for any future participant who is assigned that ART device.

**Table 1:** Articulating energy storing and returning ankle-foot devices included in the study.

Manufacturer	Device
Blatchford	Echelon
Blatchford	EchelonVT
Blatchford	Elan
Fillauer	Motion Foot
Proteor	Kinnex
Proteor	Kinterra
Ossur	Proprio
Ossur	Flex-Foot Balance
Ossur	Proflex Pivot
Hosmer	Raize
College Park	Odyssey K3
College Park	Trustep
College Park	Tribute
College Park	Venture

### Justification for Inclusion of Commercially Available ESR Ankle-Foot Devices

#### ESR Group

Many nonarticulating ESR prosthetic ankle-foot devices have different mechanical features (eg, torsion and vertical shock) that provide for specific Healthcare Common Procedure Coding System classifications, as descried in the *Prosthetic Foot Project* conducted by the American Orthotic and Prosthetic Association [31]. Although subtle mechanical differences may exist between these ankle-foot devices, studies so far have not shown any significant differences in functional outcomes between different nonarticulating ESR ankle-foot devices [32]. More specifically, published literature often reports contradictory results regarding biomechanics and function during level ground ambulation, stair negotiation, and incline and decline ambulation using these ESR ankle-foot devices [15]. Clinical consensus has also shown that patients may have individual preferences for specific nonarticulating ESR ankle-foot devices, but there is no current metric to predict this. Limiting the investigation to one or just a few nonarticulating ESR ankle-foot devices would limit the generalizability of the findings to the overall group of ESR ankle-foot devices. In addition, most patients who ultimately enroll in this investigation often have the opportunity to trial many different ankle-foot devices as part of their standard clinical care, given that prescription is not driven by reimbursement rates within VA and the DoD. For example, the limb loss care teams at VA and the DoD are not required to abide by the Medicare Functional Classification System for prosthetic prescription and can prescribe any device deemed clinically beneficial for veterans and service members. Therefore, the ESR ankle-foot device the participant enrolls with may be their foot of preference within the overall nonarticulating ESR category. Furthermore, prosthetic devices frequently change, update, or can even be taken off the market for another generation of device. Limiting the ESR ankle-foot devices may also risk not investigating the current or future prescription trends in the limb loss care clinics at all 4 study sites.

#### ART Group

The ART group includes mechanical and microprocessor-controlled prosthetic ankle-foot devices with ESR properties. These ART devices have different ankle ranges of motion, which vary from 9° to 50°. It has been reported that the average range of motion for a healthy individual with an intact lower limb is 20° to 40° of ankle plantarflexion and dorsiflexion [33]. As such, there is no published research so far regarding the ideal ankle range of motion for individuals with transtibial limb loss; thus, all commercially available ART devices that meet the study specifications will be included (Table 1). Although there are studies comparing these ART devices with solid ankle cushion heel and nonarticulating ESR ankle-foot devices, there are no current studies suggesting any differences among the devices within this group and their effect on functional performance.

#### PWR Group

Criteria for this group include the following: commercially available, articulating, and provides plantarflexion energy during terminal stance at a rate that is greater than the total energy absorbed (ie, >100%). These criteria are currently only satisfied by Empower (Ottobock Inc).

Internal analysis within all groups will be conducted at the completion of this investigation to substantiate or rebut these groupings and to determine whether there are outliers that should be classified differently for future studies.

### Research Protocol

**Figure 1:**
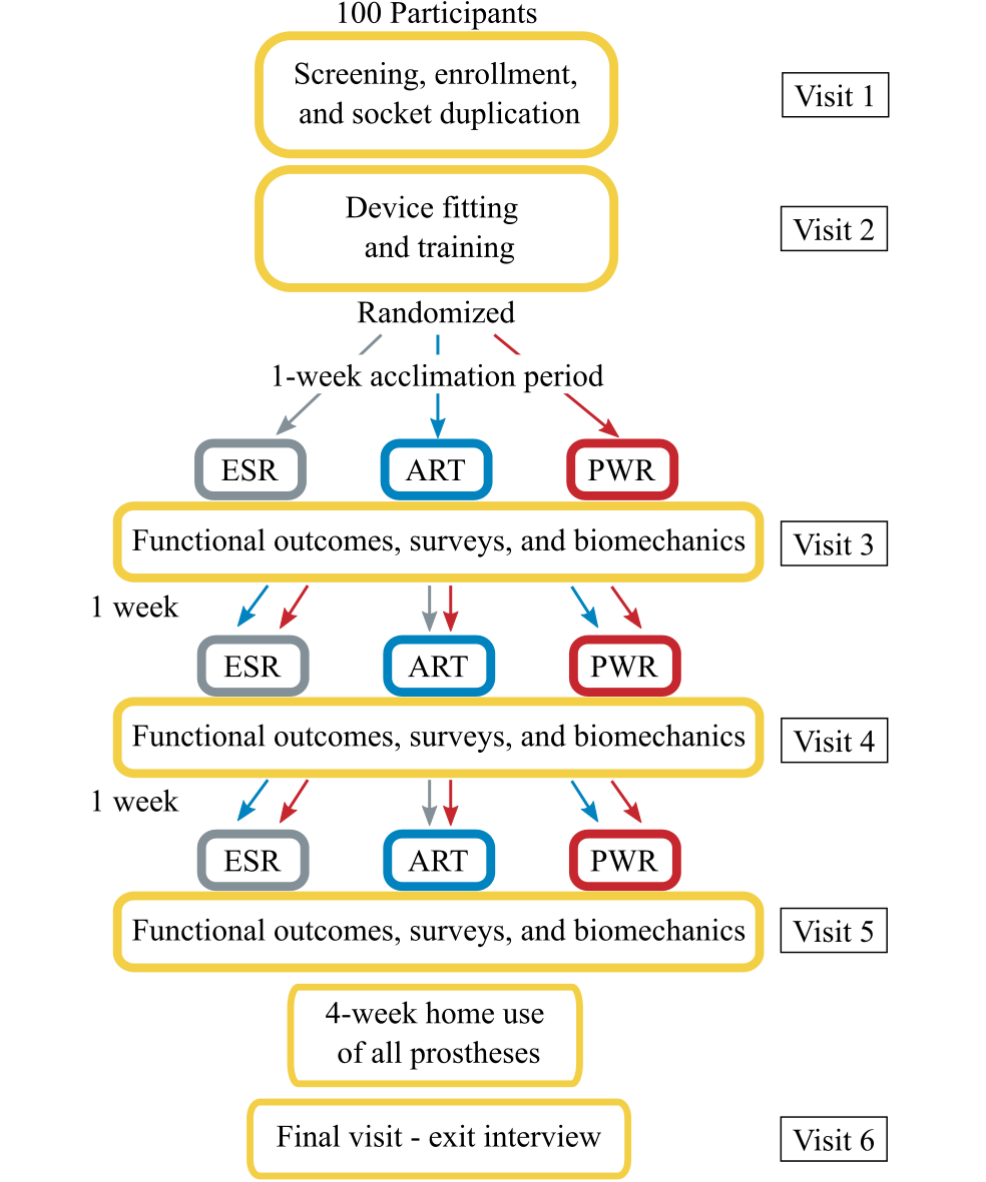
Flowchart of participant protocol visits. ART: articulating energy storing and returning; ESR: energy storing and returning; PWR: active plantarflexion energy storing and returning.

The flowchart in Figure 1 outlines the protocol procedures for each participant in this investigation. The following sections describe each participant visit.

#### Visit 1—Screening, Consent, Enrollment, and Prosthetic Socket Assessment

Participants (N=100) will be recruited from the rehabilitation and prosthetics clinics at each site. The principal investigator, site–principal investigator, research assistant, research physical therapist, or study prosthetist will meet with interested patients to discuss the investigation and obtain informed consent. Once informed consent has been obtained, participants will be screened according to the inclusion and exclusion criteria and enrolled. Demographic information will be collected, including age, sex, race, ethnicity, height, weight, limb loss etiology, employment, marital status, living situation, and time since transtibial limb loss. A baseline pain measurement using a visual analog scale (VAS) of 0 (no pain) to 100 (worst possible pain) will be performed at the first visit. Fit and comfort of each participant’s existing socket will be evaluated by the study prosthetist using standardized prosthetic guidelines. Once fit and comfort are confirmed, the study prosthetist will create a mold using plaster of Paris or alginate to fabricate 2 additional, identical prosthetic sockets. All sockets will be centrally fabricated at VANYHHS. Identical materials and prosthetic componentry used in the participant’s current socket will be used to create each additional socket to eliminate any variance in perceived weight or socket flexibility. Each additional socket will be aligned with a designated ankle-foot device according to the manufacturer’s specifications by the study prosthetist.

#### Randomization of Ankle-Foot Device Selection and Order of Evaluation

The ankle-foot devices for each participant and the order in which they will be evaluated will be block randomized using a computer-generated algorithm that randomly selects from all available devices and then assigns the order. The randomization will be performed before study enrollment, so that each combination of device order is used an equal number of times. Investigators who process and evaluate the data will be blinded to the data. Owing to the distinct differences between prosthetic ankle-foot devices, the participants and study prosthetists cannot be blinded to which foot is assigned.

#### Visit 2—Static Fitting, Dynamic Fitting, and Prosthetic Alignment

Participants will return to the prosthetic clinic for fitting of the definitive sockets and prostheses by the study prosthetist. Another subjective pain VAS measurement will be performed at the beginning of the fitting visit, before the participant returns home with an ankle-foot device. All prostheses will initially be bench aligned based on the manufacturer’s specifications for a static fitting. After successful static fitting is achieved, the dynamic alignment of each prosthesis will occur. The participant will separately ambulate with each of the prostheses, and adjustments will be made to the alignment, as needed, to achieve an optimal gait pattern, as determined by the study prosthetist and physical therapist. Dynamic fitting may require multiple visits, and participants will progress to the next part of the study protocol after an appropriate dynamic alignment has been established.

#### Device-Specific Training to Meet a Minimum Standard of Performance

After appropriate fit and alignment of each prosthesis has been established using each ankle-foot device, all participants (100/100, 100%) will undergo device-specific training by a qualified clinician to meet a minimum standard of functional performance. Overall, 2 aspects of the Functional Independence Measure will be evaluated: the ability to walk over level ground and the ability to ascend and descend stairs. To advance in the study, the participant must score 6 (modified independence) or 7 (complete independence) on the Functional Independence Measure. If the participant fails to achieve this outcome, guided rehabilitation to enhance the participant’s use of their ankle-foot device will be provided until they are able to meet the minimum standard of performance. The process of device-specific training may require multiple visits. When the minimum standard of performance is met, participants will return home with the first randomized ankle-foot device for a 1-week acclimation period.

#### Description of the Physical Therapy Plan If the Minimum Standard of Performance Is Not Met

Participants who do not meet the minimum standard of performance using a study ankle-foot device will complete a series of physical therapy training sessions lasting 30 to 45 minutes each during the training period, completing 2 sessions per week, on average. Sessions 1 and 2 will specifically focus on education, strengthening through therapeutic exercises, and early neuromuscular re-education. A home exercise program will be initiated with the participant during the initial sessions and progressed along with the program. Sessions 3 and 4 will include gait training on level surfaces. Sessions 5 and 6 will include continued progression in all areas, including multidirectional training for both neuromuscular re-education and gait. Training will conclude with sessions 7 and 8, during which the previously taught skills will be further challenged and mastered. In addition, advanced gait skills, such as ambulation on stairs and ramps, will be covered. If a participant is unable to meet the minimum standard of performance after receiving physical therapy, they will be unenrolled from the study, as determined by the study physical therapist.

#### Visits 3 to 5—Functional Assessment of ESR, ART, and PWR Ankle-Foot Devices

Visit 3 will begin after the 1-week acclimation period while using the first assigned ankle-foot device. Participants will complete the VAS pain scale and then a battery of outcome measures and surveys (outlined in the following sections). In addition, during visit 3, a subset of participants will also be randomized to undergo biomechanical gait analysis while using the first prosthesis (described in the following sections). Once visit 3 is completed, the participant will receive the second prosthesis according to the established randomization schedule, and the initial prosthesis will be returned to the study prosthetist. The participant will again complete a 1-week acclimation period. During visit 4, the participant will undergo the same pain scale and battery of outcome measures and surveys. The same subset of participants will also undergo biomechanical gait analysis using the second prosthesis. The third prosthesis will be issued at the end of visit 4, and the second prosthesis will be returned to the study prosthetist. Participants will receive the same 1-week acclimation and will complete the same pain scale and battery of outcome measures and surveys during visit 5. In addition, the same subset of participants will undergo biomechanical gait analysis while using the third prosthesis. For visits 3 to 5, a visit window of –2 to +2 days will be implemented.

#### Functional and Subjective Outcome Measures

The functional and subjective outcome measures were selected to optimize relevance to the participant population, providers, and health care systems. These measures are commonly used in prosthetic clinics and represent critical information in the functional and subjective domains needed to develop guidelines for prescription. Following the completion of the investigation, the selected outcome measures will be statistically analyzed to determine which measures are the most sensitive to changes in device type. The battery of functional outcome measures and subjective surveys collected after each acclimation period will be administered by a local research team member. Functional outcome measures include the 6-Minute Walk Test, Timed-Up-and-Go, Four Square Step Test, Amputee Mobility Predictor, Stair Assessment Index, and Hill Assessment Index. Surveys include the Prosthesis Evaluation Questionnaire, Short Form-12, and Orthotics and Prosthetics Users Survey. These measures are described in Textbox 2.

**Measures included in the study.**

**6-Minute Walk Test (6MWT)**

- 6MWT measures the distance an individual can walk in 6 minutes without help or encouragement. The 6MWT is a valid and reliable metric that correlates with physical function and functional capacity [34] and has good interrater and intrarater reliability in individuals with lower limb loss [34].

**Timed-Up-and-Go (TUG)**

- TUG measures the time taken by an individual to stand up from a standard armchair, walk 10 feet, turn, walk back to the chair, and sit down. The TUG is both reliable and valid in individuals with lower limb loss [35]. In addition, the TUG has also been used as a measure to determine risk of falling [36] and correlates well with other measures of physical functioning [37].

**Four Square Step Test**

- Four Square Step Test is a high-order, complex task assessing dynamic balance and has been validated for use in individuals with lower limb loss [38,39].

**Amputee Mobility Predictor**

- Amputee Mobility Predictor is a 21-item instrument designed to measure basic prosthetic mobility of individuals with lower limb loss [40].

**Hill Assessment Index**

- Hill Assessment Index is a rated, qualitative scale on how an individual with lower limb loss negotiates up and down an incline [41]. This will be performed on a standardized ramp (rise-to-run ratio of 1:6) with a minimum length of 4.9 m to allow for sufficient number of steps to be taken by the participant.

**Stair Assessment Index**

- Stair Assessment Index is a rated, qualitative scale for stair negotiation for individuals with lower limb loss [41]. This assessment will be performed while participants ambulate on at least 4 steps that are compliant with the Americans with Disabilities Act (17 cm high, 28 cm long, and 91 cm wide).

**Prosthesis Evaluation Questionnaire (PEQ)**

- PEQ is self-report, visual analog scale–style questionnaire for individuals with lower limb loss who use a prosthesis. It is used to evaluate the prosthesis and life with the prosthesis. It consists of 82 items with 9 functional domain scales, including ambulation, residual limb health, utility, appearance, sounds, frustration, perceived response, social burden, and well-being. The PEQ also contains items beyond the subscales, including satisfaction, pain, transfers, prosthetic care, self-efficacy, and importance [42].

**Short Form-12**

- Short Form-12 is a non–disease-specific survey that can be used to measure the relationship between physical and mental health functioning and the social determinants of health [43].

**Orthotics and Prosthetics Users Survey**

- Orthotics and Prosthetics Users Survey is a set of self-report instruments that assess functional status, quality of life, and satisfaction with prosthetic devices and services that can be used in prosthetic clinics [44].

#### Visit 6—Home-Based and Community-Based Assessment

After the completion of visits 1 to 5, participants will return home with all 3 prostheses for a period of 30 days, to assess performance and preference during home-based and community-based conditions. All 3 prostheses will be fitted with a Modus Health StepWatch 3 (Modus Health LLC), which is a research-grade instrument for long-term assessment of ambulatory activity during day-to-day life [45,46]. Capturing prosthetic performance while navigating home-based and community-based environments is a key piece of information that is yet to be effectively used in the prescription process [47]. Activity monitoring devices, such as the Modus Health StepWatch 3 used in this investigation, offer the potential to capture this critical information owing to their accuracy for those ambulating with a prosthesis [48]. Participants will also complete an activity log to document their daily activities to allow for correlation between the prosthesis used and activity. Participants are free to use each prosthesis as they choose. Ankle-foot device preference will be based upon total activity time for each prosthesis. Following 30 days of home-based and community-based use, participants will return to the prosthetic clinic to complete a closeout, guided interview to assess subjective preferences. A visit window of –5 to +5 days will be implemented for the final visit.

#### Biomechanical Analysis

In addition to completing the functional and subjective outcome measures during visits 3 to 5, a subset of 30% (30/100) of the participants will also undergo gait analysis during these visits. Biomechanical gait analysis will include kinetic and kinematic evaluation of level ground walking at 3 speeds and kinematic evaluation of incline and decline ambulation at a self-selected speed. The purpose of these sessions is to collect joint angles, forces, torques, and powers while participants use each ankle-foot device.

#### Gait Laboratory Instrumentation

Gait analysis will be performed at the Veterans Integrated Service Network 2 Biomechanics Research for the Advancement of Veteran Outcomes Laboratory at VANYHHS, the Biomechanics Laboratory at WRNMMC, and the Rehabilitation and Biomechanics Laboratory at JAHVH. The Veterans Integrated Service Network 2 Biomechanics Research for the Advancement of Veteran Outcomes Laboratory is a 1440-square foot space comprising an 11-camera motion capture system (Qualisys Inc) with 4 multiaxis force platforms (AMTI Inc). At WRNMMC, the Biomechanics Laboratory is an 1800-square foot space comprising an 18-camera motion capture system (Qualisys Inc) and 6 multiaxis force platforms (AMTI Inc). At JAHVH, the Rehabilitation and Biomechanics Laboratory is equipped with a 12-camera motion capture system (Vicon Inc) and 4 multiaxis force platforms (AMTI Inc). The systems track the positions of passive reflective markers at a rate of 120 Hz, and force platforms sample ground reaction forces at a rate of 1200 Hz. Visual3D software (C-Motion Inc) will be used for analysis of 3D motion capture data.

#### Collection of Reliable Gait Data Among Laboratories

A previous interlab reliability study was conducted among gait laboratories at the 3 military treatment facilities, which demonstrated that despite differences in marker configurations, camera systems, and examiners among the sites, reliable gait data were collected across the 3 gait laboratories [49]. A key to similarity among sites was the use of identical anatomical segment definitions for the respective gait models. To reduce variability during data collection, the 3 laboratories in this investigation will implement the key recommendations of the interlab reliability study, including the use of identical anatomical segment definitions. In addition, all 3 sites will use identical marker sets, and a single examiner at each site will conduct postprocessing of the respective data.

#### Kinematic and Kinetic Data Capture

Gait analysis will be performed after each acclimation period during visits 3 to 5. Each participant will be provided biomechanically neutral shoes that offer neither supinatory nor pronatory control. A custom, full-body passive reflective marker set will be placed on each participant, which tracks each segment independently, allowing for the accurate measurement of movements. A total of 78 markers will be placed or digitized on the head, trunk, pelvis, and extremities in the following locations: bilaterally over the positions of the medial and lateral ankle malleoli; lateral and medial condyles of the femur; tibial tuberosity; fibula head; first, third, and fifth metatarsals; anterior superior iliac spine; posterior superior iliac spine; calcaneus; lateral of the calcaneus; jugular notch; xiphoid process; spinous process of the seventh cervical vertebrae and tenth thoracic vertebrae; acromioclavicular joints; right and left scapulae; medial and lateral epicondyles of the humerus; styloid process of the radius and ulna; forearm; third metacarpal; and 4 markers on the head. Marker placements for the prosthetic limb will be matched to those of the intact leg or on centers of rotation. The cluster technique will be used to minimize the surface-to-bone displacements for the thigh-mounted, shank-mounted, and upper arm–mounted markers [50]. As such, tracking clusters will be placed bilaterally on the thigh, shank (tibial crest), and upper arm. Functional joints will also be calculated for the intact ankle and bilaterally for the knees and hips [51].

#### Data Collection Procedure

During each experimental session, participants will walk at 3 speeds across a 4-m instrumented walkway until 5 acceptable trials for each foot are completed. Trials will be considered acceptable when a foot makes full contact with a force platform. As kinetic outcome measures are speed dependent, participants will ambulate at 3 separate, controlled speeds: 1, 1.3, and 1.5 m/s. These speeds were selected to represent slow, moderate, and fast walking speeds for individuals with lower limb loss. The order of speeds will be randomized for each participant for each data collection visit. Auditory feedback will be provided to the participant by the study team to ensure that all participants walk at the targeted speed (–5% to +5%). Participants will be asked to repeat the walking trials until 5 acceptable trials for each foot at each speed are collected.

#### Incline and Decline Ambulation

Participants in this subset will also perform ramp ascent and descent to collect kinematic data during ramp ambulation. This will be performed on a standardized ramp (rise-to-run ratio of 1:6) with a minimum length of 4.9 m to allow for sufficient number of steps to be taken by the participant. Participants will be instructed to ambulate at a comfortable, self-selected walking speed. This will include 5 trials ascending and 5 trials descending the ramp.

### Data Analysis

The reflective marker positions will be digitized using motion tracking software. A 15-segment (head, trunk, pelvis, bilateral upper and lower arms, hands, thighs, shanks, and feet) rigid body model will be created based on the skin-mounted markers and functional joints. Local coordinate systems for each segment will be defined using the International Society of Biomechanics recommendations [52,53]. The data of acceptable walking trials of each participant at each speed will be processed using Visual3D. Marker data will be filtered with a 6-Hz Butterworth low-pass filter. Raw analog data will be filtered using a second-order low-pass Butterworth filter with a 25-Hz cutoff frequency. Visual3D will be used to calculate temporal-spatial values, walking speed, and lower extremity kinematics and kinetics. Inverse dynamic analysis will be applied to the kinematics of the biomechanical model and to the location, magnitude, and direction of ground reaction forces acting on the foot to calculate lower extremity joint torques and powers, including ankle, knee, and hip power of the biological and prosthetic limbs during stance and the frontal plane knee moments for the unaffected limb of participants (30/100, 30%). The resultant ground reaction force will be calculated as the magnitude of the ground reaction force vector. Several parameters, including temporal-spatial variables (eg, step lengths, stance and swing times, cadence, and stride length); peak angles; segment ranges of motion, moments, and powers for the ankles, knees, and hips during each phase of gait; and other biomechanical parameters (eg, external adduction moment rate, impulse external knee adduction moment, peak vertical ground reaction force, and peak resultant ground reaction force) will be measured and statistically compared across all conditions. Means, maximum and minimum values, ranges of motion, SEs, and coefficients of variation will be computed for the values attained per gait cycle over each trial and for each set of trials.

### Statistical Analysis

Across the study population, outcomes will be assessed using descriptive statistics (ie, means and SEs) and compared between each ankle-foot device category. Inferential statistics for ordinal data will be conducted with a repeated-measures Friedman test (α=.05) and a Dunn post hoc test at 95% CI. To identify which measures are the most sensitive to changes in ankle-foot device type, a linear mixed effects model will be used. Separate models will be run for each type of measure (ie, subjective, functional, and biomechanical), and measures that have a significant association with device type in the presence of adjusting or control variables will be determined. In the case of significance, pair-wise comparisons will be tested for significance using linear contrasts with Tukey Honestly Significant Difference or by applying Bonferroni correction. The performance of each device in each test will be ranked, and the arithmetic mode will be used to determine which device is the most suitable for each participant. Adjusting or control variables in the model will include weight, height, etiology, time since limb loss, sex, race, functional level, and current ankle-foot device.

### Development of Prediction Model

To develop evidence-based guidelines for the prescription of prosthetic ankle-foot devices, the goal of this investigation is to determine which factors and outcomes are the most predictive of optimal performance to meet patient goals. To do this, initial analyses will consist of bivariate cross-tabulations, performed using chi-square analyses. Variable selection for the multivariate model will be informed by these bivariate findings. Logistic regression will then be performed with participants’ preference of prosthesis regressed on each of several domains (eg, functional levels and outcomes, activity levels, and biomechanical parameters). The models will simultaneously control for multiple variables, and overall model predictive value will be assessed using the C-statistic. Domain-specific models (based on bivariate findings) and 1 parsimonious stepwise logistic regression model combining the domains will be created to determine whether each distinct component predicts optimal prescription outcome. Each domain-specific model will control for the identified confounding factors. The individual variables of each domain will be used as fixed predictors. Finally, stepwise multiple regression will be used to determine the factors and outcomes that represent the best predictors, which will be conducted through a series of domain-specific, forward regression models. The significant variables in the forward selection models will then be placed in a backward regression model. Significant variables from the backward regression will be considered as the strongest predictive factors and used to develop prosthetic prescription guidelines.

### Sample Size Determination and Power Analysis

Sample size determination was based on power analysis of several biomechanical, subjective, and functional measures collected during pilot investigations (Table 2). Assuming an α error rate of 5% and using within-group SDs determined from the pilot investigations, power is provided for selected parameters for a sample size of 100 participants for functional and subjective outcomes and a sample size of 30 for biomechanical parameters.

**Table 2:** Sample size determination and power analysis of selected parameters.

Parameters		Difference, mean (SD)	Power (N=100; subset of 30/100, 30%, where appropriate), %
**Biomechanical parameters**			
	Ankle range of motion (°)	2.9 (3)	95.3
	Ankle peak plantarflexion (°)	3.2 (3.6)	91.1
	Peak ankle power (W/kg)	1.7 (0.7)	99.9
	Effective foot length ratio	0.9 (0.06)	99.2
	Instantaneous radius of curvature (cm)	7.3 (9.2)	81.7
**Subjective outcome measures**			
	PEQ^a^—satisfaction	13.6 (12.4)	98.7
	PEQ—frustration	15.9 (24.7)	68.6
	PEQ—utility	8.4 (8.8)	95.5
**Functional outcome measures**			
	Steps at high activity (%)	9 (13.1)	74.4
	6-minute walk distance (m)	60 (61)	96.3

^a^PEQ: Prosthesis Evaluation Questionnaire.

## Results

The study was funded in August 2017, and data collection began in 2018. Data collection is expected to be completed before July 2023. Data analysis of the full data set is expected to begin after final data collection. Initial dissemination of results is expected in the winter of 2023, with subsequent publication of secondary analyses in 2024.

## Discussion

### Expected Outcomes and Anticipated Principal Findings

The expected outcomes of this investigation are to generate data that offer new knowledge that can be used to develop new prescription algorithms for prosthetic ankle-foot devices to improve the current clinical practice guidelines. This information will lead to clinical prescriptions that facilitate the achievement of maximal functional ability and user satisfaction for individuals with lower limb loss. Specifically, this study will help to determine the appropriate functional and biomechanical measures to support the prescription of prosthetic ankle-foot devices; correlate patient goals and subjective measures with objective data to determine the optimal prosthetic ankle-foot category to facilitate the greatest overall function for the user; and finally, develop the criteria for the appropriate prescription of nonarticulating ESR, ART, and powered plantarflexion ESR ankle-foot devices.

VA and the DoD have historically been leaders in the early adoption of advanced prosthetic technology for the clinical care of veterans and service members with limb loss. Despite the recent (and ongoing) evolution of lower limb prosthetic technology and the large number of commercially available devices, there are limited guidelines to aid the limb loss care team in prescribing appropriate prosthetic technology to achieve optimal functional ability. With the expectation of continued active lifestyles for veterans and service members with limb loss, the absence of effective, evidence-based interventions not only limits near-term outcomes but likely also increases the susceptibility for secondary physical conditions and degenerative changes over the long term. Furthermore, projected trends indicate that the overall number of amputations will increase dramatically, largely attributable to the aging population and the number of people living with dysvascular disease and diabetes [4]. As the aging veteran population advances in these diseases, this will become a major quality-of-life concern for VA, and effective outcome-based clinical practice will be necessary to decrease long-term disability and provide high quality of life. Therefore, it is the goal of this proposal to develop evidence-based lower limb prosthetic prescription guidelines for the achievement of maximal functional ability and patient satisfaction. Following the completion of this research project, the biomechanical, functional, and subjective parameters that are the most indicative in yielding the most successful and appropriate ankle-foot device prescription will have been determined. Thus, the evidence-based outcomes obtained from this research investigation can be appropriately translated into clinical practice and drive the future of clinical care and research. As future technology develops and becomes available, this protocol will help to establish the methodological framework necessary to aid in the appropriate clinical prescription of prosthetic ankle-foot devices.

### Dissemination Plan

It is expected that the outcomes and results of this investigation can become an influential source of evidence-based health care practice for prosthetic prescription that can also be incorporated in lower extremity clinical practice guidelines. Disseminating the results of this study within the DoD, VA, and civilian health care systems will be critical in changing clinical practice to affect the quality of life of individuals with transtibial limb loss. First, both VA and the DoD are integrated networks that leverage various modalities to enable the dissemination and adoption of best practices among different disciplines. Specifically for limb loss care teams, the DoD and VA Extremity Trauma and Amputation Center of Excellence leads efforts to enhance the health care of veterans and service members. Participation in the Extremity Trauma and Amputation Center of Excellence webinar series will be an excellent opportunity to present the results of this study. The webinars reach a multidisciplinary group of clinicians, scientists, researchers, and other members of the limb loss care team. This webinar series contains presentations that are broadcast and available across the entire DoD and VA health care network and occurs every other month. This forum provides an excellent platform to present the results of this study to a large, diverse audience of researchers and health care professionals in the field of limb loss care. By disseminating our results to leaders and clinicians in the field of limb loss care, we can directly influence the care provided to veterans and service members with limb loss.

To reach stakeholders in the civilian health care systems, we aim to communicate the importance of the results of this study through leading peer-reviewed publications in scientific journals and presentations at professional conferences that target clinical providers (eg, Gait and Clinical Movement Analysis Society conference, American Society of Biomechanics conference, and Military Health System Research Symposium) and by sharing our data through large data repositories.

Finally, in addition to providing a wide distribution of results through peer-reviewed publications, outcomes will be disseminated to industry leaders for improvement of their products. The DoD and VA have supported similar efforts across the prosthetics industry. Specific, relevant results provided to industry leaders can help in the evolution of lower extremity prosthetic components, which will then lead to improved devices for veterans and service members.

## Appendix

Multimedia Appendix 1 Peer review report by the US Army Medical Research and Materiel Command - Congressionally Directed Medical Research Programs - 2015 Orthotics and Prosthetics Outcomes Research Program - Prosthetics Outcomes Research Award (USA).

## Abbreviations

ART: articulating energy storing and returning
DoD: Department of Defense
ESR: energy storing and returning
IRB: institutional review board
JAHVH: James A Haley Veterans’ Hospital
PWR: active plantarflexion energy storing and returning
VA: Veterans Affairs
VANYHHS: Veterans Affairs New York Harbor Healthcare System
VAS: visual analog scale
WRNMMC: Walter Reed National Military Medical Center

## Disclaimer

The views expressed in this paper are those of the authors and do not necessarily reflect the official policy of the Departments of the Air Force, Army, Navy, Defense, and Veterans Affairs or the US Government. The identification of specific products or scientific instrumentation is considered as an integral part of the scientific endeavor and does not constitute endorsement or implied endorsement on the part of the author, Department of Veterans Affairs, Department of Defense, or any component agency.

## Data Availability

All data for this manuscript are included in this published paper. Final results will be available via a publicly available data repository.
