# Supplementary material for: Criteria for Advanced Prosthetic Foot Prescription: Rationale, Design, and Protocol for a Multisite, Randomized Controlled Trial
Source: JMIR Res Protoc. 2023 Apr 4;12:e45612. doi: 10.2196/45612 (PMC10131701; doi:10.2196/45612)
Supplement: Multimedia Appendix 1 [file resprot_v12i1e45612_app1.pdf]

Department of Defense
U.S. Army Medical Research and Materiel Command
Congressionally Directed Medical Research Programs
2015 Orthotics and Prosthetics Outcomes Research Program
Prosthetics Outcomes Research Award - Funding Level 2 - Clinical Trial
Peer Review Summary Statement

CDMRP Log Number: OP150095
Grants.gov ID Number: GRANT12039513
Meeting Dates: 01/13/2016-01/15/2016
Review Panel: Prosthetics Outcomes Research - 2

Project Duration: 36 months
Total Budget Requested: $2,487,850
Direct Costs: $2,088,314
Indirect Costs: $399,536

Title: Criteria for Advanced Prosthetic Foot Prescription
Principal Investigator: Leif Nelson
Performing Organization: Narrows Institute for Biomedical Research
Contracting Organization: Narrows Institute for Biomedical Research

Overview

The Principal Investigator (PI) of this application proposes to develop evidence-based guidelines for amputation care teams for the appropriate prescription of commercially available prosthetic foot-ankle devices in order to improve function and satisfaction. The specific aims are to (1) evaluate the differences in functional performance and subjective outcomes between nonarticulating energy-storing and returning (ESR), articulating ESR, and powered articulating ESR feet; (2) evaluate the kinematic and kinetic differences of nonarticulating ESR, articulating ESR, and powered prosthetic feet in individuals with transtibial amputation (TTA) during level-ground walking; and (3) correlate biomechanical, functional, and subjective outcome measures with user preference determined through activity monitoring and user satisfaction to develop guidelines for appropriate prosthetic prescription. The hypothesis is that articulating prostheses will have improved outcome measures over nonarticulating feet during incline/stair negotiation and that powered articulating ankles will
provide improved function and satisfaction over nonpowered articulating ankles. A prospective, multicenter, 8-week, randomized, crossover clinical trial is proposed. The intervention to be tested is prosthetic ankle-foot units (nonarticulating ESR, articulating ESR, and active [powered] plantar flexion), and the projected outcomes are functional measures (6-minute walk, Timed Up and Go [TUG], 4 Square Step Test, Amputee Mobility Predictor [AmpPro]), subjective surveys (Stair Assessment Index [SAI], Hill Assessment Index [HAI], Prosthetic Evaluation Questionnaire [PEQ], Short Form-12 [SF-12]), Orthotics and Prosthetics User Survey [OPUS]), and a full biomechanical gait analysis to collect kinematic and kinetic data.

	Average Score	Standard Deviation
**Overall Evaluation**<br>Rating Scale: 1.0 (highest merit) to 5.0 (lowest merit)	1.9<br>**(Excellent)**	0.2
**Evaluation Criteria**<br>Rating Scale: 10.0 (highest merit) to 1.0 (lowest merit)	**Average Score**	
**Research Strategy and Feasibility**	6.2	
**Impact and Military Benefit**	8.8	
**Statistical Plan**	6.8	
**Personnel**	7.3	
**Transition Plan**	8.4	

SCORED EVALUATION CRITERIA

Research Strategy and Feasibility
Average Score: 6.2

Scientist Reviewer A

The applicants hypothesize that articulating prosthetic feet will have improved outcome measures over nonarticulating feet during incline stair negotiation and that powered articulating ankles will improve function and satisfaction over nonpowered articulating ankles. The aims of the study include answering the physician's question of which foot to prescribe. The goal is to create guidelines that will include patient goals, the environment for use of prosthesis, and functional assessment to optimize prosthetic foot prescriptions. This is prospective 4-site (VA medical facilities in Seattle, New York, and Tampa as well as Walter Reed National Military Medical Center), multicenter, 8-week, randomized, crossover clinical trial. N is 120 patients. Subjects randomly receive 3 prosthetic feet (dynamic response, articulating, and powered) with duplicate sockets. Subjects will be fit and trained with each device and then separately use each prosthetic foot for 1 week of home use.

Strengths: The preliminary data and scientific rationale support the research project. The team previously evaluated the powered ankle in A Controlled Longitudinal Evaluation of the iWalk BiOM (Jason Maikos). Prosthetic ankle/feet in this study will be grouped in 3 categories: (1) nonarticulating ESR (typically the foot the subject is using); (2) articulating ESR (hydraulic with/without microprocessor); and (3) powered plantar flexion (the iWalk BiOM). The team plans to purchase the feet being tested. The feet/evaluations will be available for the duration of the proposed study. The exclusion criteria, in general, seem appropriate. The risks are described as falls and musculoskeletal. To minimize and potentially eliminate risk, all participants will be able to perform all tasks at a speed at which they feel comfortable and may refuse to complete anything they are uncomfortable completing. Privacy issues/processes for obtaining informed consent are outlined. Participants will be recruited from Physical Medicine and Rehabilitation (PMR) and the Prosthetics and Sensory Aids Service at the Veterans Affairs (VA) and the PMR and Prosthetics and Orthotics Services in the Department of Rehabilitation at Walter Reed National Military Medical Center (WRNMMC).

The PI nicely describes the population(s) of interest and strongly demonstrates access to this population with a viable plan for recruitment, consent, screening, and retention of appropriate subjects. The applicants plan to disseminate their results to the scientific and clinical community through professional conferences (Gait and Clinical Movement Analysis Society meeting, the American Physical Therapy [PT] Association Combined Sessions Meeting, the American Academy of Orthotists & Prosthetists, and American Orthotic and Prosthetic Association); targeted peer-reviewed rehabilitation publications (*Arch Phys Med Rehabil*, *J Rehabil Res Devel*, *Gait Posture*, and *Prosthet Orthot Int*); presentations at interdisciplinary meetings; and presentations to industry to optimize product development. In addition, key personnel from the Extremity Trauma and Amputation Center of Excellence (EACE) are included in the research protocol to help disseminate the findings and implement them. This study hopes to generate findings that can be developed into clinical practice guidelines and/or new prescription algorithms for prosthetic ankle/feet. The applicants hope criteria will be created to assist the amputation care teams to prescribe appropriate advanced prosthetic ankle-foot systems based on specific patient needs, goals, physical abilities, and how well the feet address the need(s) described.

Weaknesses: It is not clear why pregnant women are excluded. The applicants state that although ESR feet have different features (ie, torsion, vertical shock), no studies to date have shown a functional difference between nonarticulating ESR feet. There are huge differences between feet in the nonarticulating ESR group (Seattle-lite foot, Rush, College Park foot, Re-Flex VSP, and the Thrive). There is a big difference between hydraulic and microprocessor articulating ankles that is not adequately considered. The protocol may be biased toward the iWalk BiOM, which the team previously evaluated. (This bias may be decreased with the use of a new foot from a different manufacturer [Otto Bock] in this class; this Otto Bock foot could be included.) The most expensive foot may not provide the greatest function.

Scientist Reviewer B

The PI plans to conduct a prospective, multicentered, repeated-measures, crossover experimental design comparing 3 different prosthetic foot conditions to functional performance, subjective outcome, and user preference.

Strengths: The described method includes sufficient safeguards during all aspects of the study, including the fabrication/fit of the prosthetic intervention and protections during outcome measure testing. The documentation amply addresses all ethical and regulatory standards of human research; plans for protection of data-sharing/management are fully in place. There is ample evidence provided to support the need for this study. A major strength is the recruitment of a large sample size (N = 120) with a diverse population of subjects with TTA. As a strength to design, the PI will conduct prescreening using the Functional Independence Measure (FIM). Subjects with FIM scores of modified independence or greater will be included. Lower-level performers are not excluded, which is a strength when considering potential for those with dysvascular limb loss to have reduced functional ability. There is appropriate randomization for outcome assessment order and order for wearing each prosthetic foot. There is a sound plan to avoid differences in the prosthetic design with each condition. The prescribed foot will be randomly selected, and subjects will receive device-specific training for each foot condition to

Procurement Sensitive Document
Do not copy or distribute without CDMRP written permission.

meet the minimum FIM screening criteria before further testing. The outcome measures have demonstrated reliability and validity for the population of study. There is appropriate random assignment of a subset of 30 who will undergo gait analysis testing following the acclimation period for the 3 prosthetic foot conditions. Controlled speeds will be used to determine kinetic and kinematic differences among conditions; speeds are soundly standardized using Froude speed calculation. There is a plan to ensure gait analysis interlaboratory reliability and validity. For the third aim, the health step activity monitor coupled with an activity log, total activity time, and user satisfaction under each condition offers strong potential to determine which of the 3 prosthetic conditions is preferred by the user.

Weaknesses: There are minor to moderate weaknesses to this study. Although the foot selection will be determined by a skilled clinical prosthetist (CP), there are many choices (more than 100) of nonarticulating ESR and a few different mechanical versus microprocessor-controlled articulating ESRs. Although there is no consensus on preference or data to support any clinical difference among the feet, allowing for such a large range of feet in each study may make preference results difficult to categorize. The acclimation period between testing conditions may not be sufficient to allow for subjects to meet the minimum standard of functional performance, especially for the power ankle prosthetic foot condition. The Hill assessment instrument for stair/inclines assessment does not have demonstrated intrarater reliability. The implementation of 3 different quality-of-life (QOL) instruments, the SF-12, and OPUS, in addition to the PEQ may not be necessary and may serve only to inflate results for preference. The standardized ramp exceeds Americans with Disabilities Act guidelines for percent incline, so lower-level subjects may not be able to complete this component. There are possible ceiling effects for subjects with functional skills well beyond the FIM 3 screening requirements, particularly affecting the AmpPro and TUG scores.

Bioethicist Reviewer

Strengths: This research study is about the effects of a powered ankle-foot prosthesis and device-specific PT on function and pain for individuals living with transfemoral limb loss. More specifically, it is looking at the criteria for advanced prosthetic foot prescription. This is a sound study. It focuses on 3 classes of prosthetic devices. The recruitment strategy appears appropriate.

Weaknesses: The data and safety–monitoring plan needs to be sufficiently described. It is possible that observations of the subjects may result in information regarding injuries, discomforts, or problems not anticipated at the outset. It is unclear if the researchers will provide information to the clinicians after the study regarding the devices most appropriate for individual participants. The consent form should tell participants whether they will be given a prosthetic after their active participation. The consent form is not adequately clear on this issue. The consent form could provide more information about data and safety monitoring that will be done. The application discusses activity monitoring but does not adequately describe monitoring that might lead to changes in exclusion criteria, for example.

Procurement Sensitive Document
Do not copy or distribute without CDMRP written permission.

Discussion Notes

There was significant discussion about the research strategy and feasibility of this application. The scientific rationale for this study is strong. In general, the research strategy appears fine, and the outcome measurements are fairly comprehensive in scope. However, many panel members were concerned that the 1 week of home use before evaluation may be too short. There are a very large number of nonarticulating ESR feet, whereas the articulating ESR feet class has many fewer feet. Although there may be no data to support any clinical difference between the feet in the nonarticulating feet class, this still was a concern for some panel members. In addition, there was some discussion about the subjects' baseline fitness level as a confounding variable and concerns about a ceiling effect for subjects with a high mobility before the study. Although the study does try to control to this to some degree, some panel members felt that prestudy activity could be assessed in more detail and more substantially taken into consideration. Differences of opinions were not entirely resolved during the discussion.

Impact and Military Benefit
Average Score: 8.8

Scientist Reviewer A

Strengths: The goal is to ultimately create guidelines that will include patient goals, patient environment for use of prosthesis, and functional assessment to drive prescription of the most appropriate device to allow the veterans or service members (SMs) to thrive in the present day as well as over the course of their lifetime. If successful, the outcomes determined from this study could lead a shift in the paradigm of care for individuals with TTAs to achieve the most optimal functional ability and overall satisfaction.

Weaknesses: The applicants may overstate what this project will accomplish. The applicants state that (1) the absence of evidence-based prescription may make this population of SMs and veterans with amputations more susceptible to increased secondary physical conditions and degenerative changes such as osteoarthritis earlier in life, and (2) veterans and SMs with nontraumatic amputations are at even higher risk of comorbidities associated with a sedentary lifestyle due to premorbid health conditions of individuals with dysvascular amputation. It is not clear how these statements will be supported by this study.

Scientist Reviewer B

Strengths: The PI effectively illustrates the need for this study in the military population. He particularly notes the increasing number of servicemen and veterans living longer with lower-limb amputation, as many are acquiring limb loss in combat at a young age. For those SMs and veterans who lose a lower limb at a younger age as well as in later life, there is an increased risk for developing long-term health complications such as joint dysfunction and pain. Successful outcomes from this study may allow for more immediate rigorous guidelines in the prescription of prosthetic foot-ankle systems that have potential to reduce these medical risks. There are potential long-term benefits for optimizing functional performance and enhancing user satisfaction if the created prediction model for prosthetic foot-ankle function is validated.

Procurement Sensitive Document
Do not copy or distribute without CDMRP written permission.

Another major strength of this study is the sample size and inclusion of active-duty or retired SM (or dependent) subjects from 4 large VA and military medical centers. This large representative sample will adequately allow for greater generalization of study results, including that of the civilian sector. Overall, the study is not novel in design; however, the recruitment of a large sample size could potentially provide a vast array of relevant clinical data for determining prosthetic foot preferences and function.

Weaknesses: No weaknesses were noted.

Consumer Reviewer

Strengths: The purpose of this study is to develop guidelines to aid the amputation care team to prescribe appropriate prosthetic technology to achieve optimal functional ability. By maximizing functional ability, the patient may enjoy increased activity with decreased discomfort. Long-term secondary physical conditions and degenerative changes could also be minimized, leading to a better QOL for the patient.

Weaknesses: No weaknesses were noted.

Discussion Notes

The potential impact of a study such as this was viewed as high. Evidence-based guidance to allow more appropriate prosthetic foot-ankle system prescriptions could be a benefit to all persons with amputations. However, it is not clear if an appropriate prosthetic foot-ankle system prescription alone will significantly alter the physical activity level of a person with an amputation.

Statistical Plan
Average Score: 6.8

Scientist Reviewer A

Strengths: Study design, N, and comprehensive functional measures (6-Minute Walk Test, TUG, 4 Square Step Test, AmpPro, SAI, HAI, PEQ, SF-12, OPUS, and a subset of participants [n = 30] who will undergo a full biomechanical gait analysis to collect kinematic and kinetic data during the level ground and incline/decline walking for each foot/period) will provide a strong evaluation of the feet/subject function. The final phase will allow users to try out all 3 prostheses at the same time to choose when and why they use or do not use each prosthetic foot.

Weaknesses: It is not clear how compliance will be monitored. For example, how use of the devices will be monitored is not adequately described (diary, accelerometer, or other device).

Scientist Reviewer B

Strengths: There is clearly described and accurately applied statistical testing to address all hypotheses for the repeated-measures design of this study. Statistical measures include linear mixed models for ANOVA, correlational analyses, and stepwise multiple regression to address

Procurement Sensitive Document
Do not copy or distribute without CDMRP written permission.

the 3 leading hypotheses. All post hoc testing procedures are clearly outlined. For the second major aim, linear mixed-effects models will be effectively used to identify significant differences in gait temporal-spatial and biomechanical variables and to examine the relationship between intact knee peak forces and the various prosthetic foot conditions as well as to determine the association between prosthetic peak ankle power under different foot conditions. Correlational analyses will allow for examination of subject preference in relation to use with an activity monitor over a 30-day period. Finally, there is a sound plan to implement bivariate cross-tabulation and chi square to create a prediction model for prosthetic prescription.

Weaknesses: There is 1 potential moderate weakness to this study. Adequate power calculations for all hypotheses are not included. Power for Aim 2, the gait kinetic and kinematic analyses, is presented but is below the generally acceptable standard of 0.8 (80%). Since 120 subjects are being recruited for this study, it might be warranted to include a larger number of subset subjects to increase power. Another minor weakness noted is that it is unclear about the rationale for inputting HAI and SAI results for both functional and subjective outcomes. There is potential that use of many different ESR feet may not adequately allow for determination of differences between feet and function or user preference.

Biostatistician Reviewer

Strengths: The methods appear appropriate. The strategy is generally well thought out. The sample size calculation is generally well explained and justified.

Weaknesses: In terms of the statistical model and data analysis strategy, potentially missing data/dropout is not adequately considered. The effect of multiple testing in case of multiple outcome measures does not appear to be adequately addressed. The predictive models considered come from a very narrow class and may be improved over by statistical (machine) learning techniques. In model selection, the heuristic search (stepwise regression) may be unwarranted unless the model space is very large. The investigators propose to develop predictive models but do not adequately address validation. In terms of sample size calculation, sufficient specific details affecting the calculation (such as variance components) are not provided.

Discussion Notes

There was significant discussion on the statistical plan. In general, the strategy and sample-size calculation to be used are well described. A model for association appears to be feasible, although the plan to create prediction models is not clear, and some panel members were concerned that this might not be achievable. It appears that the sample size may be based on a single-outcome model; however, multiple outcomes are measured. The subset sizes may become too small. The statistical analysis plan for Aim 3 was viewed as limited. The degree of concern about the statistical plan varied among panel members.

Personnel
Average Score: 7.3

Scientist Reviewer A

The PI, Leif Nelson, DPT, ATP, CSCS, of New York, New York (VA) is currently an assistant chief of clinical care for the congressionally mandated Extremity Trauma and Amputation Center of Excellence. Leif Nelson earned a DPT in physical therapy in 2002 from New York University. Collaborators include Jason Maikos, PhD, director, VISN 3Gait and Motion Analysis Laboratory, and Jeffrey T. Heckman, DO, Medical Regional Amputee Center, Seattle VA.

Strengths: Dr Nelson has a first-rate multidisciplinary team (physicians, therapist, engineers) of investigators. The investigators' accomplishments demonstrate their ability to accomplish the proposed work.

Weaknesses: The effort of a senior statistician could be reduced, moving the routine work to junior statistics consultants. The PI, Dr Nelson, is not yet well established in dissemination of his research. The positions for research coordinator and prosthetic technician are not yet determined.

Scientist Reviewer B

The PI, Leif Nelson, received his doctoral degree in physical therapy from New York University and is currently assistant chief of clinical care for the Extremity Trauma and Amputation Center of Excellence.

Strengths: Dr Nelson has excellent clinical experience working with military members and civilians with lower-limb amputation. There are major strengths in this multidisciplinary team for conducting grant-funded research. The PI and Coinvestigators, Dr Maikos and Mr Christopher Fantini, have a record of success in conducting research as a team. Dr Maikos has expertise in gait analyses and ample experience with the iWalk (BiOM) power ankle. The Coinvestigator PT/prosthetist (CP), Mr Fantini, has over 16 years of clinical practice experience and experience in the fit/function of the BiOM power ankle; he has submitted research with the power ankle. The key personnel from the additional sites, Dr Alison Pruziner, Dr Christopher Dearth, Dr Jeffrey Heckman, and Dr Samuel Phillips have extensive research experience and records of publication in research related to the proposed study. The PT PI at WRNMMC, Dr Pruziner, has many years of experience working with SMs and veterans with limb loss and will effectively be able to implement outcome assessments. The level of effort is appropriate for all key personnel and the additional members: research assistant, study coordinator, statistician, and prosthetic technician.

Weaknesses: The PI, Dr Nelson, is not yet well established in dissemination of his current research through publication in refereed journals. The positions for research coordinator and prosthetic technician are not yet determined.

Biostatistician Reviewer

Dr Paul Kolm is the consulting statistician on the project. Dr Kolm is director of biostatistics at Christiana Care Health System, research professor of medicine at Thomas Jefferson Medical College, and director of the BADER Consortium Biostatistics Core.

Procurement Sensitive Document
Do not copy or distribute without CDMRP written permission.

Strengths: Dr Kolm is appears to be a highly experienced biostatistician.

Weaknesses: The following are minor weaknesses. It does not appear that there was a previous collaboration between the PI and the statistician. The proposed analyses are fairly standard, and most may be completed by a junior consulting statistician. The PI may likely benefit more by reducing the effort of a senior statistician and moving the routine work to junior statistical consultants.

Discussion Notes

There was significant discussion about the personnel for this project. In general, this was considered a strong multidisciplinary team. However, there were some concerns that the PI and a Co-I, Dr Nelson and Dr Maikos, have no significant journal publications on research studies of gait biomechanics or prosthetics. Dr Nelson has some reviews and case studies, but no significant research studies are evident. Dr Maikos has 3 research publications on rat spinal cord injury research but no significant publications on human gait biomechanics or prosthetics that have been published. Differences of opinions were not entirely resolved during the discussion.

Transition Plan
Average Score: 8.4

Scientist Reviewer A

Strengths: The funding strategy described to bring the outcome(s) to the next level of development and/or delivery to the military or civilian market is appropriate. Results from this study will be used to apply for continued funding to further refine prescription guidelines. The DOD offers an array of opportunities that challenge the scientific community to optimize the care to SMs and veterans with limb loss. The next phases of this investigation fall within the focus area of many of these opportunities, including the Prosthetics Outcomes Research Award, US Army Medical Research and Materiel Command (USAMRMC) broad agency announcement, and the Peer Reviewed Orthopaedic Research Program. Furthermore, as the aging veteran population advances in dysvascular disease and diabetes, this will become a major financial and QOL concern for the VA, and effective outcomes-based clinical practice will be necessary to decrease long-term disabilities associated with prosthetic usage. As such, the Rehabilitation Research and Development Service for the VA is interested in funding investigations that can potentially reduce this future burden on the VA system. The schedule and milestones for bringing the outcome(s) to the next level of development are appropriate and feasible. Intellectual property is not an issue. The study is using commercially available feet. Appropriate collaborations are well described. Results from this study will be used to apply for continued funding to further refine prescription guidelines.

Weaknesses: No weaknesses were noted.

Scientist Reviewer B

Strengths: The strategy (and funding) for conducting the current study is effective for accomplishing the objectives of this study. The overall outcome of this current study is to create

a major source of evidence-based clinical practice for prosthetic prescription of foot/ankle components in individuals with TTA. There are clearly outlined plans for dissemination of the current research findings to key DOD and VA leaders in the field, and a there is a plan in place to reach out to industry leaders for improvement in products (foot-ankle components). The PI has well-established collaborative partnerships with Coinvestigators and facility resources to carry out this study. In addition, the schedule/milestones for achievement of this study are realistic and feasible.

Weaknesses: The risk analyses for cost and/or future sustainability, outside of conducting further research, are not clearly outlined in the application. As a result, the long-term plans are relatively unknown.

Discussion Notes

In general, the Transition Plan was viewed as appropriate. There are significant funding resources for follow-up studies. However, there was some concern that the transition of this research into the clinic is not clear and may be difficult. Differences of opinions were not entirely resolved during the discussion.

UNSCORED EVALUATION CRITERIA

Environment

Scientist Reviewer A

Sites include the New York VA, Walter Reed National Military Medical Center, the Tampa VA, and the Seattle VA. The VA space, equipment, and other resources described in the application will be available to successfully execute this investigation. The data management between sites is well outlined: A 1-day training kickoff meeting will be held prior to study initiation to review and train research staff. Additional training will take place to review the biomechanical gait analysis among sites. Monthly videoconferences among all sites will be conducted.

Scientist Reviewer B

The scientific environments at all 4 sites have provided full support of the proposed research study for recruitment and study implementation. The VA New York Harbor Healthcare System (NYHHS), Walter Reed National Military Medical Center (WRNMMC), VA Puget Sound Health Care System (VAPSHCS), and Tampa VA James A. Haley Veterans Hospital are fully supportive of this research application. The space and equipment resources at each facility are more than sufficient to conduct all outcome assessments, and the VA NYHHS and Walter Reed National Military Medical Center (WRNMMC) have gait and motion analysis laboratories that offer state-of-the-art data collection for the subset of subjects. The PI and collaborators have sufficient access to all of the needed resources at the facilities and adequate ability to teleconference as needed. Each of the 4 sites has prosthetic and orthotic services that are fully staffed and have equipped laboratories for the design, fabrication, and fitting of advanced

Procurement Sensitive Document
Do not copy or distribute without CDMRP written permission.

prosthetic technology. There is superb support of PT services in both facilities, and if PT is warranted during the study, implementation will be overseen by the PI, Dr Nelson.

Budget

Scientist Reviewer A

The overall budget, $2,491,350.63, is lower than anticipated. Several of the Coinvestigators are VA employees, so no salary is required.

Scientist Reviewer B

The budget seems appropriate for the number of key personnel, additional personnel, equipment, and resources required for this multicenter study. There is sufficient justification for budget needs for the prosthetic component, including the BiOM (power-ankle). Subaward costs from collaborators at Walter Reed National Military Medical Center, VA Puget Sound VA Medical Center, and Health Services Research & Development (HSR&D)/Rehabilitation Research and Development (RR&D) Center of Innovation on Disability and Rehabilitation Research at the Tampa VA are included and appropriate for support.

Application Presentation

Scientist Reviewer A

The application clearly outlines the protocol: No details are spared (data management among sites, prosthetic fabrication, testing, PT, and gait motion analysis). However, there are grammatical errors.

Scientist Reviewer B

On the Application Form SF-424, item 1, the PI checks that this is a changed/corrected application; in item 8, the checked box is New (not revised). The Lay Abstract may be overly simplified. There are 1 or 2 run-on sentences and vague statements within the body of text (Technical Abstract and Transition Plan). There is a major heading missing in procedure: The PI mentions parts 2 to 4 of study under Specific Aim 1, but the purpose of identifying the parts of study is unclear. Overall, these are relatively minor issues that do not negatively impact the overall strength of the application.

Procurement Sensitive Document
Do not copy or distribute without CDMRP written permission.
